# Correlation between Very Short and Short-Term Blood Pressure Variability in Diabetic-Hypertensive and Healthy Subjects

**DOI:** 10.5935/abc.20180020

**Published:** 2018-02

**Authors:** Karina R. Casali, Beatriz D. Schaan, Nicola Montano, Daniela Massierer, Flávio M.F. Neto, Gabriela H. Teló, Priscila S. Ledur, Marilia Reinheimer, Graciele Sbruzzi, Miguel Gus

**Affiliations:** 1 Instituto de Cardiologia - Fundação Universitária de Cardiologia (IC/FUC), Porto Alegre, RS - Brazil; 2 Universidade Federal do Rio Grande do Sul, Porto Alegre, RS - Brazil; 3 L. Sacco Hospital - University of Milan, Milan - Itália;; 4 Divisão de Cardiologia - Hospital de Clínicas de Porto Alegre, Porto Alegre, RS - Brazil; 5 Serviço de Cardiologia - Hospital de Clinicas de Porto Alegre, Porto Alegre, RS - Brazil

**Keywords:** Hypertension, Diabetes Mellitus, Type 2, Autonomic Nervous System, Blood Pressure Monitoring, Ambulatory

## Abstract

**Background:**

Blood pressure (BP) variability can be evaluated by 24-hour ambulatory BP monitoring (24h-ABPM), but its concordance with results from finger BP measurement (FBPM) has not been established yet.

**Objective:**

The aim of this study was to compare parameters of short-term (24h-ABPM) with very short-term BP variability (FBPM) in healthy (C) and diabetic-hypertensive (DH) subjects.

**Methods:**

Cross-sectional study with 51 DH subjects and 12 C subjects who underwent 24h-ABPM [extracting time-rate, standard deviation (SD), coefficient of variation (CV)] and short-term beat-to-beat recording at rest and after standing-up maneuvers [FBPM, extracting BP and heart rate (HR) variability parameters in the frequency domain, autoregressive spectral analysis]. Spearman correlation coefficient was used to correlate BP and HR variability parameters obtained from both FBPM and 24h-ABPM (divided into daytime, nighttime, and total). Statistical significance was set at p < 0.05.

**Results:**

There was a circadian variation of BP levels in C and DH groups; systolic BP and time-rate were higher in DH subjects in all periods evaluated. In C subjects, high positive correlations were shown between time-rate index (24h-ABPM) and LF component of short-term variability (FBPM, total, R = 0.591, p = 0.043); standard deviation (24h-ABPM) with LF component BPV (FBPM, total, R = 0.608, p = 0.036), coefficient of variation (24h-ABPM) with total BPV (FBPM, daytime, -0.585, p = 0.046) and alpha index (FBPM, daytime, -0.592, p = 0.043), time rate (24h-ABPM) and delta LF/HF (FBPM, total, R = 0.636, p = 0.026; daytime R = 0,857, p < 0.001). Records obtained from DH showed weak positive correlations.

**Conclusions:**

Indices obtained from 24h-ABPM (total, daytime) reflect BP and HR variability evaluated by FBPM in healthy individuals. This does not apply for DH subjects.

## Introduction

Blood pressure (BP) variability results from the interplay between external environmental stimuli, vascular system, and biological autonomic regulation of circulation.^1^ Abnormalities in BP variability, evaluated by continuous intra-arterial ambulatory BP monitoring, are associated with poor outcomes in normotensive and hypertensive subjects.^2-4^ Noninvasive methods such as finger BP measurement (FBPM) are good alternatives to invasive BP monitoring, as they are accurate non-invasive estimates of beat-to-beat radial BP, providing data that can estimate very short-term BP variability.^5,6^ In addition, beat-to-beat records allow the extraction of information regarding heart rate (HR) variability that is directly related to cardiac autonomic control impairment^7,8^ and associated with poor outcomes in both general^9^ and diabetic populations.^10^ However, due to practical and economic reasons, this method cannot be routinely used in the evaluation of outpatients.

The development of noninvasive 24-hour ambulatory BP monitoring (24h-ABPM), with multiple readings throughout day and night, has made short-term BP variability estimate through several indices possible.^11^ However, there are major differences between BP variability obtained from beat-to-beat records and that obtained by 24h-ABPM. Besides the duration of the series-very short- (FBPM) or short-term (24h-ABPM)-, BP series obtained by FBPM allows studying beat-to-beat variability, while 24h-ABPM series are sampled every 10-15 minutes within 24 hours.^6^ While non-invasive beat-to-beat methods allow detecting fast oscillations resulting from inter-beat variations, it is inefficient to access very slow waves in short series; 24h-ABPM, in turn, detects slow variations only.^12,13^ As both methods provide information about BP signals originating from the same cardiovascular system, a correlation between oscillatory components of overlapping bands obtained from FBPM and 24h-ABPM is expected; however, studies on the association between BP variability evaluated by 24h-ABPM indices and target organ damage have shown contradictory results.^4,14^

Our report was aimed to compare three different parameters of short-term BP variability in 24h-ABPM, with very short-term BP variability measured by indices obtained from the FBPM in healthy subjects and in a population at high cardiovascular risk comprised of diabetic hypertensive subjects.

## Methods

### Study design and population

This cross-sectional study was conducted at the outpatient clinic of the Hospital de Clínicas de Porto Alegre (Porto Alegre, RS, Brazil), a tertiary teaching hospital, and Instituto de Cardiologia do Rio Grande do Sul, Fundação Universitária de Cardiologia, from January 2009 to December 2012. The study was approved by the Ethics Committee of both Institutions (nº 0469.0.001.000-08 and 4313/09, respectively), which is accredited by the Office of Human Research Protections as an Institutional Review Board, in agreement with the principles outlined in the Declaration of Helsinki. After protocol approval, all subjects signed a written informed consent for participation. Adult patients of both genders, aged 18-65 years, and with hypertension and type 2 diabetes mellitus were invited to participate (DH group). Control group (C) consisted of healthy subjects, that is, without diagnosis or medication for hypertension and diabetes.

### Clinical evaluation

Patients underwent demographic and clinical baseline data collection. Diabetes mellitus was defined by two fasting plasma glucose ≥ 126 mg/dl or use of antidiabetic agents or personal history of diabetes. Blood pressure was measured with an office aneroid sphygmomanometer and the mean values were estimated after an two measures on average. The cuff size was selected according to arm circumference. Hypertension was defined by mean blood pressure ≥ 140/90 mmHg or use of antihypertensive medication. After baseline data collection, subjects were randomly assigned to evaluations, being first submitted to 24h-ABPM or to FBPM. The interval between the two examinations was of no more than 15 days. 

### Short-term blood pressure variability evaluation (24h-ABPM)

All individuals were submitted to a 24h-ABPM in a usual working day, using a monitor (Spacelabs 90207, Spacelabs, Redmond, WA). Measurements were obtained every 15 minutes from 7 a.m. to 11 p.m., and every 20 minutes from 11 p.m. to 7 a.m. to complete 24 hours of the period studied. Cuff size was selected according to subjects' arm circumference.^14^

Based on the results of 24h-ABPM, the mean 24-hour systolic (SBP) and diastolic (DBP) blood pressures were calculated for each patient. Three different parameters of SBP variability were calculated: 1) time-rate index (rate of change in SBP over time in mmHg/min, defined as the first derivative values of SBP by time); 2) coefficient of variation of systolic BP within 24 hours (SD/mean pressure x 100%); and 3) mean of standard deviation of 24-hour systolic BP. The time-rate index allows the calculation of angular coefficients' sum and aims to measure how fast or slow and in which direction SBP values change. The measure was calculated using the following formula, where r is the rate of BP variability over time (considering the differences between BP measurements at each time interval) and N is the number of recordings:^15^

$$R=\left|\bar{r}\right|=\frac{\sum_{i=1}^{N-1}\left|\mathrm{ri}\right|}{N-1}$$

In addition, considering circadian variations of BP and possible differences between daytime and nighttime 24h-ABPM parameters, data were divided into daytime and nighttime according to patients' reports and were also analyzed separately, considering both periods. Circadian behavior differences were calculated by subtracting nighttime from daytime values for each parameter.

### Very short-term blood pressure variability evaluation (FBPM)

Blood pressure was recorded continuously, on a beat-to-beat basis, using the FINAPRES system (Ohmeda 2300, Monitoring Systems, Englewood, CO, USA).^16^ In this method, the pressure wave can be continuously monitored by a sensor placed on the patient's non-dominant middle finger, detecting small oscillations only. The experimental protocol had measurements at two different stages: ten minutes at rest in a sitting position and ten minutes after standing-up maneuver (sympathetic activation).

The BP signal was digitized by the CODAS system (Computer Operated Data Acquisition Software; DATAQ, Instruments, AKRON, OH, USA), sampling at 1 kHz and analyzed for each condition. Pulse interval (PI) tachogram and systolic arterial (SA) systogram series were constructed through the algorithm of Windaq/DATAQ, which identifies systolic peaks from BP waves. Systogram and tachogram series were analyzed by spectral analysis (frequency domain analysis) using an autoregressive model, applied to stationary intervals, which were selected in each segment condition. The stationarity of each time series was tested as previously reported.^17^ Short-term BP and HR variabilities were evaluated based on systogram and tachogram analyses, respectively.

In humans, the frequency domain analysis considers three distinct bands: high frequency (HF), which includes the interval between 0.15 and 0.4 Hz; low frequency (LF) between 0.04 and 0.15 Hz; and very low frequency (VLF), lower than 0.04 Hz.^18,19^ The same analysis was applied to tachogram series. Among parameters obtained by frequency domain analysis, LF and HF components are distinguished by physiological significance. They are mainly related to sympathetic and parasympathetic cardiac modulations, respectively; the relation between them-LF/HF index-is related to sympathetic-vagal balance;^20^ and the absolute powers of LF and VLF components are predominantly related to vascular sympathetic modulation and to renin-angiotensin system modulation on SBP, respectively.^1^ The alpha index was obtained from the square root of the ratio between the LF component of tachogram and systogram when coherence, assessed by cross-spectral analysis, exceeded 0.5 in these bands^21^ and expressed spontaneous baroreflex sensitivity. All series were analyzed by a trained researcher who was also blinded to conditions and subjects.

Delta indices were calculated for HR variability (HRV), LF/HF index and LF component of BPV, using variable values before (rest) and after standing-up maneuver (sympathetic activation, SA) for normalization, as follows:

$$\mathrm{Delta}=\frac{\mathrm{SA}-\mathrm{REST}}{\mathrm{REST}}$$

These indices had been previously proposed in order to quantify autonomic responses to standing-up maneuver.^22,23^

### Biochemical measurements

Venous blood samples for biochemical measurements were drawn after 12-hour fasting. Plasma glucose was determined by the glucose oxidase method, serum creatinine by Jaffé's reaction, and glycated hemoglobin (HbA1c) by ion-exchange HPLC (Merck-Hitachi L-9100 HbA_1c_ analyzer; Merck, Darmstadt, Germany). Serum cholesterol and triglycerides were measured by enzymatic-colorimetric methods (Merck Diagnostica, Darmstadt, Germany; Boehringer Mannheim, Buenos Aires, Argentina), and HDL cholesterol by a homogeneous direct method (autoanalyzer, ADVIA 1650). Low-density lipoprotein (LDL) cholesterol was calculated using Friedewald's formula.^24^

### Statistical analyses

Data are expressed as mean ± standard deviation (SD) or medians and interquartile intervals, according to normality plots with tests and percentages. Pearson's chi-square, unpaired Student's t-test, Mann-Whitney rank sum test, two-way repeated measures ANOVA or Friedman repeated measures analysis of variance on rank, *post hoc* Student-Newman-Keuls were used when variables were compared between groups, as indicated. The correlation between the different indices obtained by 24h-ABPM and by FBPM were analyzed by the Spearman's correlation coefficient. Correlations were considered for discussion only if they were statistically significant and represented large-effect sizes, as defined by a correlation coefficient of 0.50 or higher.^25^ All statistical analyses were performed using the SPSS statistical software package version 17.0 for Windows (SPSS Inc., Chicago, IL, USA). Statistical significance was set at p < 0.05. 

## Results

Twelve healthy subjects (C) and 73 diabetic-hypertensive patients (DH) were selected; all C and 51 DH had complete data from 24h-ABPM and FBPM. Controls were 51.7 ± 8.1 years old and 50% were men; DH were 57.3 ± 8.1 years-old, 12% were men, 54.9% had their office BP well-controlled (< 130/80 mmHg) and 35.8% had good metabolic control (HbA1c < 7.0%). Clinical characteristics are shown in Table 1.

**Table 1:** Clinical characteristics of controls (C) and diabetic-hypertensive (DH) subjects.

Variables	Controls (n = 12)	Diabetic-hypertensive (n = 51)	p
Age (years)	51.6 ± 4.4	57.3 ± 8.1	0.011
Male gender	6 (50.0)	11 (20.8)	0.065
BMI (Kg/m^2^)	23.5 ± 2.3	30.5 ± 4.2	< 0.001
Office SBP (mmHg)	116.0 ± 8.2	139.2 ± 17.2	< 0.001
Office DBP (mmHg)	77.0 ± 5.0	80.9 ± 11.9	0.086
Duration of diabetes (years)	-	6.9 (3.0-10.0)	
Fasting plasma glucose (mg/dL)	-	156.5 ± 55.1	
HbA1c (%)	-	8.2 ± 2.0	
Total cholesterol (mg/dl)	-	181. 2 ± 32.6	
HDL cholesterol (mg/dl)	-	42.6 ± 13.2	
Triglycerides (mg/dl)	-	180.5 (132.8 - 248.5)	
Creatinine (mg/dl)	-	0.82 ± 0.2	
Microalbuminuria (> 17 µg/min)	-	14 (27.4)	

BMI: body mass index, SBP: systolic blood pressure, DBP: diastolic blood pressure, HbA1c: glycated hemoglobin, HDL: high-density lipoprotein; FBPM: Short-Term Blood Pressure. Continuous variables are expressed as mean ± standard deviation or median and interquartile range (p25-p75) and percentiles. Categorical variables are expressed as number (%). Comparisons were tested by Pearson's χ2 test and Student's t test.

**Figure 1:**
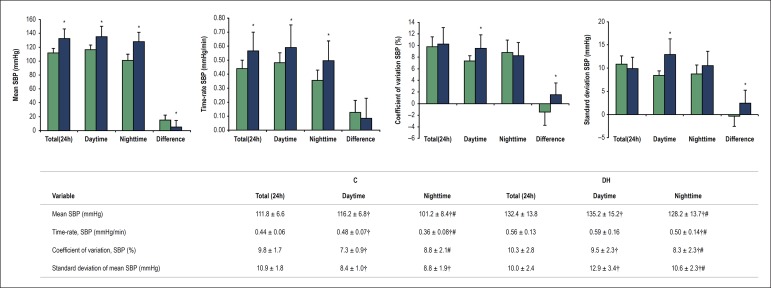
Short-term BP variability (24h-ABPM). Data are shown as mean ± SD. SBP, systolic blood pressure. Analysis using 24-hour records (total) and divided into daytime and nighttime periods. Graph shows a comparison between controls (C, green bars) and diabetic-hypertensive (DH, blue bars) groups, also including circadian behaviour differences, calculated by subtracting nighttime from daytime values (Difference, last bars of each graphic). * p < 0.05 vs. C, Student's t-test. Table reports all values, compared by Friedman repeated measures analysis of variance on rank, post hoc Student-Newman-Keuls. † p < 0.05 vs. total period; # p < 0.05 vs. daytime period.

Short-term BP variability (24h-ABPM) results are displayed in Figure 1. There were differences among the indices obtained from total, daytime, and nighttime periods for both C and DH groups, confirming the expected circadian variations and justifying the division into periods. Comparisons between C and DH groups are represented by the bar graph, which shows that higher SBP and time-rate obtained from SBP in DH group for all periods evaluated. The mean of the standard deviation and the coefficient of variation of 24-hour SBP were different between C and DH in daytime only. Circadian behavior differences, calculated by subtracting nighttime from daytime values, show a lower reduction of mean SBP at night in DH patients as compared to controls. The differences obtained for the coefficient of variation and mean of standard deviation of 24-hour systolic BP were positive in DH, negative in C, and different between groups.

Very short-term BP variability and HR variability (FBPM) results obtained by spectral analysis are displayed in Table 2. As expected, BP was higher in DH *vs.* C at rest and after standing-up maneuver. Heart rate variability, LF component of HRV, and alpha index were lower in DH *vs.* C. The standing-up maneuver, applied to induce sympathetic activation, resulted in differences for all HR variability components, showing the expected response to this maneuver in both groups. However, BP variability did not change after the maneuver in DH subjects, and the alpha index (spontaneous baroreflex sensitivity) was lower at rest and after the maneuver in this group when compared to controls.

**Table 2:** Very short-term blood pressure variability and heart rate variability (FBPM) at rest and after standing-up maneuver.

Variable	C (n = 12)		DH (n = 51)		P		
	At rest	Standing up	At rest	Standing up	Group	Condition	Interaction
Mean BP (mmHg)	114.6 ± 23.6	112.6 ± 18.2	129.4 ± 17.7	123.8 ± 21.6	0.019	0.494	0.147
Very short-term BPV (mmHg^2^)	21.57 ± 12.20	37.17 ± 16.12*	25.27 ± 20.08	25.23 ± 23.04	0.504	0.058	0.047
LF component of BPV (mmHg^2^)	1.80 ± 1.13	13.84 ± 11.93	4.59 ± 6.02	9.26 ± 10.61	0.879	< 0.001	0.060
HF component of BPV (mmHg^2^)	1.40 ± 1.57	3.73 ± 3.02*	3.44 ± 3.46#	2.25 ± 1.64*	0.538	0.323	< 0.001
Mean HR (bpm)	66.2 ± 9.3	77.8 ± 8.7*	70.8 ± 11.9	77.2 ± 13.9*	0.419	< 0.001	0.016
HRV (s^2^)	1.47 ± 1.71	1.07 ± 0.78	0.73 ± 0.79	0.45 ± 0.42	0.005	0.010	0.658
LF component of HRV (nu)	40.77 ± 16.84	61.48 ± 16.90	31.08 ± 21.07#	40.69 ± 23.62	0.014	< 0.001	0.339
HF component of HRV (nu)	50.65 ± 14.79	30.07 ± 15.33	49.91 ± 22.35	36.19 ± 20.48	0.842	< 0.001	0.464
LF/HF index	0.99 ± 0.76	3.33 ± 3.16	1.09 ± 1.54	2.69 ± 4.13	0.902	0.001	0.744
Alpha index (ms/mmHg)	15.04 ± 9.75	6.43 ± 5.24*	8.24 ± 7.70#	4.72 ± 3.89*	< 0.001	< 0.001	0.003

Data are shown as mean ± SD. HR: heart rate, HRV: heart rate variability, BP: blood pressure, BPV: BP variability, LF: low frequency component, HF: high frequency component.

* p < 0.05 vs. rest condition;

# p < 0.05 vs. C; FBPM: Short-Term Blood Pressure. Two-way repeated measures ANOVA. Post hoc Student-Newman-Keuls.

**Figure 2:**
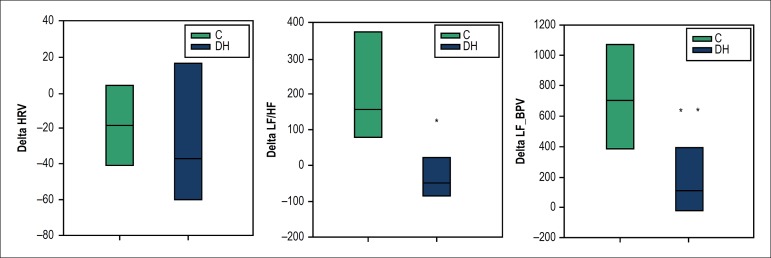
Autonomic response to standing-up maneuver as evaluated by delta indices: delta HRV (heart rate variability), delta LF/HF (low frequency/high frequency) and delta of LF of blood pressure variability (LF_BPV), calculated from HR variability, LF/HF index and LF component of BPV, respectively. Box plots (median, 25 and 75% interquartile intervals) showing comparison between controls (C, blue bars) and diabetic-hypertensive (DH, green bars) groups. * p < 0.001 and ** p = 0.009 vs. C, Mann-Whitney rank sum test.

Autonomic response to standing-up maneuver assessed by delta indices (Figure 2) had a lower response for LF/HF ratio in DH group as compared to C group. Changes in delta HRV and delta_LF/HF variability (BPV) were not different.

Correlations between very short- and short-term BP variabilities are shown in Tables 3 (C group) and 4 (DH group). In C group, some correlations were found at rest, and some after the standing-up maneuver. At rest, standard deviation of 24-hour systolic BP (24h-ABPM) was positively correlated with the LF component of BP variability (FBPM) in 24-hour evaluation; the coefficient of variation (24h-ABPM) was negatively correlated with total BP variability and alpha index (FBPM) during daytime. After standing-up maneuver, time-rate (24h-ABPM) was positively correlated with the LF component of BP variability (FBPM, 24 hours, and daytime). Time-rate (24h-ABPM) was correlated with delta_LF/HF (FBPM, 24 hours, and daytime). In DH group, although some correlations were statistically significant, none of them represented large-effect sizes (correlation coefficient of 0.50 or higher). Moderate-effect sizes (correlation coefficient near 0.50) were shown for total BP variability (24h-ABPM), coefficient of variation, and standard deviation (FBPM, 24 hours, and daytime). There was no correlation between short-term (24h-ABPM) and very short-term variability (FBPM) parameters, considering delta indices for DH subjects.

**Table 3:** Correlation between very short- (FBPM) and short-term (24h-ABPM) BP variability parameters - Control group.

	24-hour			Daytime			Nighttime		
	Time-rate	Coefficient of variation	Standard deviation	Time-rate	Coefficient of variation	Standard deviation	Time-rate	Coefficient of variation	Standard deviation
**AT REST**									
Total BPV R	0.236	-0.042	-0.007	-0.247	-0.585	-0.315	0.555	0.210	0.301
(P)	0.461	0.897	0.983	0.439	0.046	0.319	0.061	0.513	0.341
LF component BPV R	0.373	0.538	0.608	0.370	0.445	0.559	0.021	0.343	0.438
(P)	0.233	0.071	0.036	0.236	0.147	0.059	0.948	0.276	0.155
Alpha index R	-0.127	-0.357	-0.322	-0.483	-0.592	-0.524	0.290	-0.056	0.063
(P)	0.695	0.255	0.308	0.111	0.043	0.080	0.361	0.863	0.846
**AFTER SYMPATHETIC ACTIVATION (Standing‑up maneuver)**									
Total BPV R	0.418	0.350	0.427	0.138	-0.172	0.084	0.180	0.392	0.424
(P)	0.176	0.265	0.167	0.670	0.594	0.795	0.575	0.208	0.170
LF component BPV R	0.591	0.322	0.385	0.649	0.329	0.413	-0.074	0.315	0.263
(P)	0.043	0.308	0.217	0.022	0.296	0.183	0.819	0.319	0.409
Alpha index R	-0.116	-0.392	-0.322	-0.346	-0.312	-0.322	0.191	-0.503	-0.270
(P)	0.720	0.208	0.308	0.271	0.324	0.308	0.552	0.095	0.397
**DELTA INDEXES (AT REST/AFTER SYMPATHETIC ACTIVATION)**									
Delta_HRV R	-0.056	-0.070	-0.105	0.353	0.081	-0.042	-0.541	-0.168	-0.340
(P)	0.862	0.829	0.746	0.261	0.803	0.897	0.070	0.602	0.280
Delta_LF/HF R	0.636	0.000	0.217	0.857	0.385	0.392	-0.233	-0.126	0.088
(P)	0.026	1.000	0.499	0.000	0.216	0.208	0.466	0.697	0.787
Delta LF_BPV R	0.299	-0.091	-0.077	0.282	0.011	-0.035	-0.170	-0.035	-0.217
(P)	0.346	0.779	0.812	0.374	0.974	0.914	0.598	0.914	0.498

BPV: blood pressure variability, LF: low frequency, LF­_BPV: LF component of BPV; FBPM: Short-Term Blood Pressure. Statistic correlation expressed as correlation coefficient (R) and significance (P), obtained by Spearman's test.

**Table 4:** Correlation between very short- (FBPM) and short-term (24h-ABPM) BP variability parameters - DH group.

	24-hour			Daytime			Nighttime		
	Time-rate	Coefficient of variation	Standard deviation	Time-rate	Coefficient of variation	Standard deviation	Time-rate	Coefficient of variation	Standard deviation
**AT REST**									
Total BPV R	0.03	0.280	0.240	0.210	0.261	0.286	-0.036	0.026	0.103
(P)	0.486	0.046	0.090	0.152	0.064	0.042	0.807	0.857	0.471
LF component BPV R	0.330	0.356	0.347	0.332	0.185	0.259	0.234	0.278	0.337
(P)	0.022	0.010	0.013	0.021	0.194	0.066	0.110	0.048	0.016
Alpha index R	-0.371	-0.250	-0.264	-0.420	-0.171	-0.222	-0.158	-0.151	-0.220
(P)	0.009	0.076	0.062	0.003	0.229	0.117	0.283	0.289	0.122
**AFTER SYMPATHETIC ACTIVATION (Standing‑up maneuver)**									
Total BPV R	0.192	0.403	0.413	0.269	0.447	0.486	0.033	0.029	0.176
(P)	0.191	0.003	0.003	0.065	0.001	0.000	0.826	0.841	0.218
LF component BPV R	0.140	0.274	0.283	0.156	0.166	0.245	0.042	0.090	0.134
(P)	0.341	0.052	0.044	0.290	0.244	0.083	0.777	0.532	0.349
Alpha index R	-0.359	-0.206	-0.263	-0.405	-0.098	-0.192	-0.172	-0.306	-0.336
(P)	0.012	0.146	0.063	0.004	0.493	0.177	0.243	0.029	0.016
**DELTA INDEXES (AT REST/AFTER SYMPATHETIC ACTIVATION)**									
Delta_HRV R	0.054	0.018	-0.011	-0.003	-0.045	-0.058	0.055	0.106	0.059
(P)	0.714	0.901	0.938	0.985	0.754	0.688	0.711	0.460	0.679
Delta_LF/HF R	-0.015	-0.152	-0.099	-0.037	-0.215	-0.190	0.088	0.097	0.083
(P)	0.922	0.291	0.492	0.807	0.134	0.186	0.557	0.501	0.568
Delta LF_BPV R	-0.162	-0.105	-0.077	-0.210	-0.093	-0.070	-0.069	-0.070	-0.097
(P)	0.271	0.464	0.590	0.152	0.515	0.623	0.643	0.623	0.500

BPV: blood pressure variability, LF: low frequency, LF_BPV: LF component of BPV; DH: Diabetic-Hypertensive. Statistic correlation expressed as correlation coefficient (R) and significance (P), obtained by Spearman's test

## Discussion

BP and HR variabilities were assessed in healthy and diabetic-hypertensive individuals by two well-known methods-24h-ABPM and FBPM-, seeking potential concordance between results of each method, which was indeed observed. Correlations between indices of BP variability (time rate with LF component BPV, standard deviation with LF component BPV, and coefficient of variation with total BPV and alpha index) and indices of HR variability (time rate with delta_LF/HF) were high and significant in controls. On the other hand, few moderate correlations were observed in diabetic-hypertensive patients only after sympathetic activation.

As expected, there were differences between 24h-ABPM indices obtained in total, daytime, and nighttime periods because of the well-known circadian variations of BP levels^26,27^ which occurred in both healthy and diabetic-hypertensive subjects. This leads us to conclude that data were adequately collected. Moreover, periods division showed differences between groups only when data collection included the day period, in accordance with previous reports.^28,29^

Additionally, indices obtained from FBPM had lower HRV, LF component of HRV, and alpha index (at rest and after standing-up maneuver) in DH *vs.* C group. This finding suggests the presence of autonomic neuropathy in the diabetic population, as expected and previously demonstrated by evaluating similar indices.^30,31^

In controls, correlations between very short- and short-term BP variability were present with FBPM data at rest and after the standing-up maneuver, but only when daytime data were included. This probably occurs because both methods are evaluating BP signals in similar situations, as 24h-ABPM provides data obtained mostly during routine activities in standing-up position (mean duration of nighttime period ~6.9h). The most significant correlations were those between time-rate index (24h-ABPM) and LF component of BP variability and delta_LF/HF (FBPM); also between the coefficient of variation (24h-ABPM) and between total BPV and the alpha index in all periods that included daytime data. The time-rate index obtained by 24h-ABPM (24-hour or daytime period) in healthy individuals is expected to reflect what the reference standard (FBPM) would show, considering LF component of BP variability and delta_LF/HF.

The weak correlations observed between 24h-ABPM and FBPM indices in the diabetic-hypertensive group depict a very different pattern, which is certainly related to their disease. Moreover, there was no correlation between short-term variability parameters and delta indices. These correlations are weak even though four times more patients were evaluated, which would show significant correlations if they in fact existed. We cannot exclude that one or both methods employed may provide false results for this specific population once FBPM, for example, depends on attaining good BP signals, and quality of such information was not good because of vascular disorders common to this population.^32^ Therefore, we do not recommend 24h-ABPM to estimate very short-term BP variability parameters based on short-term variability indices for diabetic-hypertensive individuals.

Currently, the evaluation of BP variability across the several indices that can be obtained from 24h-ABPM or home blood pressure monitoring is not recommended by guidelines,^14,33^ for predicting cardiovascular risk, or as additional goal for antihypertensive therapy, because literature has no consensus on these issues.^4,14,34,35^ It is possible that evidence available is not strong enough to support this use because the tools used are not so reliable. We suggest that equations derived from the 24h-ABPM measurement for non-diabetic subjects would be useful for risk prediction, but not for diabetic-hypertensive patients. It is unknown, though, whether this pattern occurs in hypertensive-only populations. The use of BP variability reduction as a new target to explore in further intervention trials related to hypertension should only be considered after this information is validated.

Considering the high prevalence of autonomic neuropathy in diabetes,^36,37^ and characteristic changes of this complication detected in the diabetic-hypertensive group (circadian behavior differences, lower spontaneous baroreflex sensitivity, HR variability and lower responses to stand-up in the LF/HF ratio vs. controls), we attributed to this complication some of the differences observed in other indices between groups. The standing-up maneuver is usually applied to induce sympathetic activation in very short-term BP variability evaluation, and in fact it induced the expected cardiac autonomic response for many indices in controls, but not for most of them in diabetic-hypertensive individuals.

Taking clinical characteristics of diabetic-hypertensive subjects into account and bearing in mind that the sample studied was obtained from a tertiary center, many patients were not adequately monitored (BP and metabolic control), indicating a high-risk group. Perhaps in this high-risk population, variability found in 24h-ABPM or other home BP evaluation methods may not successfully qualify higher cardiovascular risk beyond absolute systolic or diastolic BP, as previously described.^34,38^ Also, the age differences found could, at least partially, overestimate the differences between groups, and therefore configure a limitation of this study.

## Conclusions

In summary, short-term BP variability measured by time-rate index, standard deviation or coefficient of variation in 24h-ABPM are correlated with LF component BPV and delta_LF/HF obtained from FBPM in nondiabetic individuals. Such findings should be evaluated in further cohort studies adequately designed for this purpose, also seeking relations with hard outcomes. This correlation was not well established in diabetic-hypertensive subjects. Some indices obtained from FBPM for diabetic subjects are promising tools for the diagnosis of diabetic autonomic neuropathy. Considering a standard reference for the diagnosis of autonomic neuropathy, these indices and cutoff values should be evaluated in further studies adequately designed for this purpose.
